# Heightened mitochondrial respiration in CF cells is normalised by triple CFTR modulator therapy through mechanisms involving calcium

**DOI:** 10.1016/j.heliyon.2024.e39244

**Published:** 2024-10-11

**Authors:** H.H. Jarosz-Griffiths, L.R. Caley, S. Lara-Reyna, S. Savic, I.J. Clifton, M.F. McDermott, D.G. Peckham

**Affiliations:** a Leeds Institute of Medical Research, University of Leeds, United Kingdom; b Institute of Microbiology and Infection, University of Birmingham, United Kingdom; c Leeds Institute of Rheumatic and Musculoskeletal Medicine, University of Leeds, United Kingdom; d Department of Clinical Immunology and Allergy, St James's University Hospital, United Kingdom; e Department of Respiratory Medicine, Leeds Teaching Hospitals NHS Trust, Leeds, United Kingdom

**Keywords:** Cystic fibrosis, Metabolism, Elexacaftor/tezacaftor/ivacaftor, Mitochondrial reactive oxygen species, Mitochondria, CFTR, Calcium

## Abstract

**Background:**

Cystic fibrosis (CF) is associated with increased resting energy expenditure. However, the introduction of elexacaftor/tezacaftor/ivacaftor (ETI) has resulted in a paradigm shift in nutritional status for many people with CF, with increase body mass index and reduction in the need for nutritional support. While these changes are likely to reflect improved clinical status and an associated downregulation of energy expenditure, they may also reflect drug-induced alterations in metabolic perturbations within CF cells. We hypothesise that some of these changes relate to normalisation of mitochondrial respiration in CF.

**Methods:**

Using wild-type (WT) and F508del/F508del CFTR human bronchial epithelial cell lines (HBE cell lines) and baby hamster kidney (BHK) cells we examined the impact of ETI on cellular metabolism. We monitored mitochondrial respiration, using Seahorse extracellular flux assays and monitored mitochondrial reactive oxygen species (mROS) and intracellular calcium levels by flow cytometry.

**Results:**

Increased mitochondrial respiration was found in HBE cell lines and BHK cells expressing CFTR F508del/F508del when assessing basal, maximal, spare respiratory capacities and ATP production, as well as increased mitochondrial ROS generated via forward electron transport. ETI significantly decreased basal, maximal, spare respiratory capacity and ATP production to WT levels or below. Calcium blocker, BAPTA-AM normalised mitochondrial respiration, suggesting a calcium-mediated mechanism. ETI decreased intracellular calcium levels in CF cells to the same extent as BAPTA-AM, highlighting the importance of calcium and chloride in mitochondrial respiration in CF.

**Conclusions:**

CF cell lines exhibit increased mitochondrial respiration, which can be downregulated by ETI therapy through mechanisms involving calcium.

## Highlights

• Mitochondrial respiration and ROS were measured pre/post ETI in CF and WT cells.
• Mitochondrial Respiration and Reactive Oxygen Species were elevated in CF.
• ETI and calcium blockers decrease Mitochondrial Respiration and ROS.
• ETI and calcium blockers decreased intracellular calcium flux in CF cells.
• ETI may decrease Mitochondrial Respiration and ROS via changes in intracellular calcium.

## 1 Introduction

Alterations in mitochondrial function were first identified as early as the 1970s in skin fibroblasts isolated from people with CF. Feigal and Shapiro documented heightened mitochondrial calcium uptake, oxygen consumption, and changes in the kinetics of mitochondrial NADH dehydrogenase. Similarly, Von Ruecker and colleagues observed elevated electron transfer activities in CF cells, but found no change in the activity of complex I in CF cells [[1], [2], [3], [4], [5]]. Dysregulation in mitochondrial calcium metabolism was also reported in the CF phenotype in a range of cell types including skin fibroblasts [4], bronchial goblet cells, kidney cells, and lymphocytes [6], and more recently in airway epithelial cells [7] and neutrophils [8]. In all these cell types, the intracellular calcium concentration was increased compared to non-CF cells, suggesting that functional CFTR regulates Ca^2+^ homeostasis.

Increased cytosolic and mitochondrial Ca^2+^ levels can drive adenosine triphosphate (ATP) production by modulation of the Krebs cycle enzymes, pyruvate dehydrogenase, isocitrate dehydrogenase and α-ketoglutarate [9]. Enhanced ETC activity can cause electrons to leak out and interact with oxygen to produce reactive oxygen species (ROS). Increased cellular ROS and mitochondrial oxidative stress has been reported in CFTR-deficient human lung cells [10], and high levels of oxidative stress are associated with increased apoptosis in CFTR-mutated tracheal and pancreatic cells [11].

We have recently shown increased ETC activity and oxygen consumption rate (OCR) in CF M1 macrophages relative to healthy controls [12]. In contrast, studies have reported reduced complex I activity in both CF airway epithelial and murine CF colonic epithelial cells [13,14], as well as reduced expression of the MT-ND4 gene, which could potentially impact overall electron chain activity. The MT-ND4is gene encodes nicotinamide adenine dinucleotide hydride (NADH) dehydrogenase subunit 4 (MTND4), a protein which is required for the correct assembly and function of complex I [14]. Defective complex I function is also associated with excessive mitochondrial ROS production and has been implicated in the pathogenesis of several mitochondrial diseases [15]. Correction of mitochondrial dysfunction with a CFTR modulator, VX-809, restored ETC activity and reduced mitochondrial ROS in impaired CF bronchial epithelial cells [13].

Most people with CF have increased resting energy expenditure (REE), driven in part by chronic infection, inflammation, and exocrine pancreatic dysfunction [16]. This increase in REE may also reflect functional changes in cellular metabolism. Treatment with highly effective modulators such as elexacaftor/tezacaftor/ivacaftor (ETI), has been associated with increased body mass index (BMI) in people with CF. This is presumed to be the result of improved clinical stability confounded by an increased appetite and nutritional intake [17]. A recent study tentatively suggested that energy intake decreased post ETI, despite elevation in BMI, suggesting that other mechanisms may be at play [18]. We hypothesise that some of these changes may relate to changes in mitochondrial respiration in CF. The aim of this study was to assess mitochondrial respiration in cells with CF-causing mutations and the response to ETI therapy.

## 2 Materials and methods

### 2.1 Cell line culture

Human bronchial epithelial cell lines (HBE cell lines), NuLi-1 (wild-type, N1) (ATCC CRL-4011), CuFi-1 (CFTR F508del/F508del, C1) (ATCC CRL-4013) were obtained from ATCC (UK), The cell lines were authenticated on the August 6, 2015 and then re-authenticated by the ATCC supplier on September 10, 2024, using short tandem repeat (STR) analysis to determine the DNA profile. Cell lines were originally generated using the pNUT expression plasmid with methotrexate (500 μm) selection, followed by clonal selection and screening using CFTR specific antibody mAb M3A7 as described by Haardt M. et al., 1999. Cell lines were tested for mycoplasma contamination on the same date using the indirect Hoechst DNA strand method, direct agar culture method, and PCR-based assay, all of which were reported negative. Cells were grown on Cell + surface flasks (Sarstedt, Leicester, UK) and were cultured in airway epithelial cell growth medium (Sigma-Aldrich, Gillingham, Dorset). Baby hamster kidney (BHK) cells expressing human WT CFTR (WT) or human F508del/F508del CFTR were a generous gift from MD Amaral (University of Lisboa, Portugal), Cells had been authenticated within three years of experiments (prior to being sent on February 18, 2020) and screened mycoplasma negative. Cells were cultured in DMEM/F-12 medium containing 5 % (v/v) foetal bovine serum (FBS) and 500 μM of methotrexate to ensure continued expression of each construct. Cell lines were cultured in an incubator at 37 °C with 5 % CO_2_. Cells were grown to 80–90 % confluency and then detached, using (0.05 %) Trypsin-EDTA (Thermo Fisher Scientific, Loughborough, UK), for no longer than 15 min in the incubator for the HBE cell lines, and no longer than 5 min at room temperature for the BHK cells.

### 2.2 Cell treatments

To partially correct CFTR channel activity, cells were pre-treated with triple CFTR modulator combination, elexacaftor (3 μM), tezacaftor (18 μM) and ivacaftor (1 μM), and (ETI) for 48 h or with the equivalent concentration of dimethyl sulfoxide (DMSO) (control) for 48 h at 37 °C. Drug concentrations were selected based on a recent study in human primary airway epithelial and BHK cells expressing F508del as well as the dose used by Keating et al. to describe the *in vitro* effect of modulators on CF airway epithelial cells [19,20].

To inhibit ETC complexes, cells were pre-treated with either rotenone (0.5 μM; 2 h) for complex I, dimethyl malonate (DMM) (1 mM; 3 h) for complex II or antimycin A (10 μM; 2 h) for complex III. Intracellular calcium levels were reduced using cell permeable calcium chelator, BAPTA-AM (20 μM, 30 min).

### 2.3 Glycolytic and mitochondrial assays (extracellular flux analyser)

BHK cells were seeded at a density of 0.25 x 10^5^ and HBE cell lines were seeded at a density of 0.5 × 10^5^ in XF96 tissue-culture microplates (Agilent, Cheadle, UK), previously coated with CellTak (Corning), according to the manufacturer's instructions. After a minimum of 1 h adherence for BHK cells, or overnight for HBE cell lines, cells were pre-incubated with ETI modulators or DMSO control for 48 h. On the day of the experiment, cells were incubated with BAPTA-AM, as indicated in the cell treatments section. Following incubations, cells were washed twice with XF DMEM medium, containing 10 mM Glucose, 2 mM L-glutamine and 1 mM sodium pyruvate for Mito stress Test then incubated for an hour in a non-CO^2^ incubator. Next, the basal levels of oxygen consumption rates (OCR) were measured on a Seahorse XFe 96 Extracellular Flux Analyzer (Agilent). Once basal OCR measurements were obtained, cells were stimulated with oligomycin (1 μM), FCCP (1 μM), and rotenone/antimycin A (0.5 μM), following the instructions stated in the XF Cell Mito Stress Test Kit (Agilent, Cheadle, UK) To ensure consistent cell numbers across cell lines and treatments, following analysis, cells were fixed with 70 % ethanol for 10 min at room temperature. Subsequently, the cells were incubated in a solution of 3 μM DAPI diluted in PBS for 15 min and then washed three times in PBS. Fluorescence was measured on a plate reader with excitation set at 370 nm and emission at 460 nm. A range of metabolic parameters were calculated from the data, as shown in Table 1.

**Table 1:** Metabolic calculations.

**Parameter**	**Equation**
**Basal respiration**	(measurement before oligomycin stimulation) – (rate measurement after rotenone/antimycin A stimulation)
**Proton leak**	(minimum rate measurement after oligomycin stimulation) – (minimum measurement after rotenone/antimycin A stimulation)
**Maximal respiration**	(maximum rate measurement after FCCP stimulation)– (minimum rate measurement after rotenone/antimycin A stimulation)
**Spare respiratory capacity**	(maximal respiration) – (basal respiration)
**ATP production**	(basal respiration) – (minimum rate measurement after oligomycin stimulation)

**Mito Stress test**	

### 2.4 Mitochondrial ROS

To establish the source and direction of ROS produced by the mitochondrial ETC, inhibitors of complex I (rotenone, ROT), II (dimethyl malonate, DMM) and III (antimycin A, ANTI A) were employed. Complex I and complex III, especially complex I, are the main sites of ROS production in mitochondria. ROS can be generated in the matrix during the transfer of electrons from NADH to CoQ in complex I. Rotenone inhibits the electron transfer to CoQ and increases ROS production through forward ETC activity [21]. Cells were seeded onto six well plates at a density of 0.5 x 10^6^ for BHK cells, or 1 x 10^6^ for HBE cell lines, and allowed to adhere overnight. Cells were pre-treated with ETC complex inhibitors, ETI or BAPTA-AM, as described above. Intracellular mROS in HBE cell lines and BHK cells were measured and analysed by flow cytometry, using Invitrogen’s MitoSOX assay (M36008). MitoSOX was resuspended in DMSO and diluted 1 mM stock in PBS. Cells were incubated with 500 μL of 1 μM MitoSOX solution (2 % FBS) for 30 min. The cells were then washed twice with PBS (2 % FBS), and finally resuspended in PBS (2 % FBS) for analysis. Cells were stained with DAPI, and single live cells were gated with 30,000 events captured per sample.

### 2.5 Intracellular calcium measurements

Cells were seeded onto 96 well plates at a density of 10 x 10^5^ for BHK cells or 50 x 10^5^ for HBE cell lines and allowed to adhere prior to being cultured for 48 h with ETI modulators or DMSO control. On the day of the experiment, cells were cultured with BAPTA-AM, as described above. Cells were washed once with cell specific culture media, then incubated in the dark with 1 μM Fura-2, AM (high affinity intracellular calcium indicator) and 10 % (w/v) F-127 plutonic acid (Invitrogen, UK) at 37 °C for 1 h. Cells were rinsed twice with standard bath solution (SBS) which contains 134 mM NaCl, 5 mM KCl, 1.2 mM Mg_2_Cl, 1.5 mM Ca_2_Cl, 8 mM glucose and 10 mM HEPES, pH 7.4 before being incubated in BAPTA-AM (20 μM) diluted in SBS at 37 °C for 30 min. Following incubation, the fluorescence was measured using a Flex-Station II384 (Molecular Devices), with excitation parameters (Lm1 340 nM, Lm2 380 nM) and emission parameters (Lm1 510 nm, Lm2 620 nm) being set; a ratio of Lm1/Lm2 was calculated and an endpoint reading taken after 5 min recording.

### 2.6 Statistical analysis

All analyses were performed using GraphPad Prism v 9. Graphs show three independent experiments with 3–6 technical replicates in each experiment for each cell line and each condition unless otherwise stated. An unpaired *t*-test or a one-way ANOVA statistical test, with Tukey’s or Dunnett’s multiple comparison, when calculating variance between samples with a normal distribution (p values ∗≤0.05, ∗∗≤0.01, ∗∗∗≤0.001 and ∗∗∗∗≤0.0001). A p < 0.05 was considered significant.

## 3 Results

### 3.1 Oxygen consumption rate is increased in CF cell lines

**Fig. 1:**
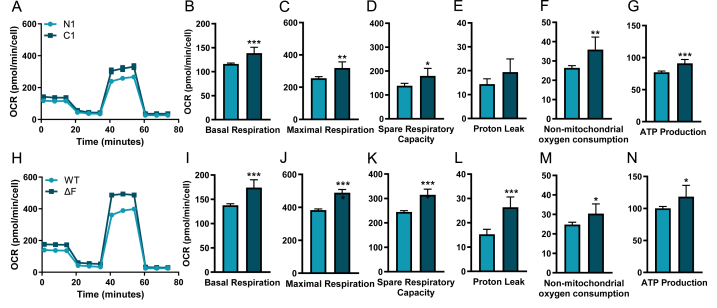
Oxygen consumption rate (OCR) is increased in cells expressing F508del/F508del CFTR relative to WT CFTR controls. OCR levels were measured in the wild-type HBE cell lines NuLi1 (N1), and CF HBE cell lines CuFi1 (C1) (A-G). OCR levels were measured in WT BHK (WT CFTR) and ΔF BHK (F508del/F508del CFTR) (H-N). Basal respiration (B, I), maximal respiration (C, J), spare respiratory capacity (D, K), proton leak (E, L), non-mitochondrial oxygen consumption (F, M) and ATP production (G, N) were all measured. These graphs show three independent experiments with 3–6 technical replicates in each experiment for each cell line and each condition. All values were calculated as described in the methods. All data are presented as mean ± 95 % CI. (∗). Statistical comparisons were performed using an unpaired *t*-test, ∗p < 0.05, ∗∗p < 0.01, ∗∗∗p < 0.001, ∗∗∗∗p < 0.0001.

Using Seahorse extracellular flux assays we showed that CuFi1 (C1) HBE cell lines and ΔF BHK (ΔF) cells have increased OCRs relative to their WT CFTR counterparts (NuLi1 (N1) HBE cell lines and WT BHKs (WT)) (Fig. 1). There was a significant increase in basal respiration (C1, p = 0.0008; ΔF, p < 0.0001), maximal respiration (C1, p = 0.0039; ΔF, p < 0.0001), spare respiratory capacity (C1, p = 0.0279; ΔF, p < 0.0001), ATP production (C1, p = 0.0003; ΔF, p = 0.0467) and non-mitochondrial oxygen consumption (C1, p = 0.0038; ΔF, p = 0.0420) in both C1 HBE cell lines (Fig. 1B, C, D, G) and ΔF BHK cells (Fig. 1I, J, K, N) relative to WT controls. Proton leak was also increased in ΔF BHK cells (p = 0.0005) and a small but upward trend in C1 HBE cell lines (p = 0.1843) (Fig. 1 L, E respectively). These data suggest that the F508del/F508del CFTR mutation in C1 and ΔF cells elicits an overall increase in mitochondrial activity relative to WT controls, in the absence of infection.

### 3.2 Mitochondrial ROS is increased in CF via forward electron transport

**Fig. 2:**
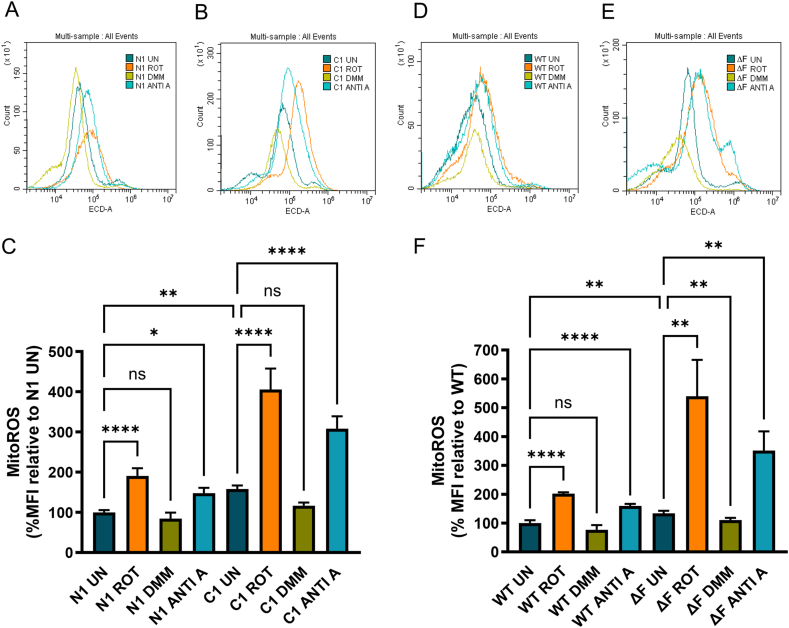
Mitochondrial ROS is increased in HBE cell lines and BHK cells expressing F508del/F508del CFTR. Mitochondrial ROS was measured in N1 HBE cell lines and C1 HBE cell lines (A, B, C), WT BHK and ΔF BHK (D, E, F). Cells were incubated in the presence/absence of rotenone (ROT), dimethyl malonate (DMM), or antimycin A (ANTI A), and then incubated with MitoSox Red and mitochondrial ROS (mROS) measured by flow cytometry. mROS is represented as % mean fluorescence intensity (MFI) relative to WT untreated controls (C, F). These graphs show three independent experiments with 2 technical replicates in each experiment for each cell line and each condition. All values were calculated as described in the methods. All data are presented as mean ± 95 % CI. (∗). Statistical comparisons were performed using a one-way ANOVA followed by Tukey’s multiple comparison test, ∗p < 0.05, ∗∗p < 0.01, ∗∗∗p < 0.001, ∗∗∗∗p < 0.0001.

Elevated levels of mROS were observed in C1 HBE cell lines (p = 0.0019, Fig. 2A and B) and ΔF BHK cells (p = 0.0014, Fig. 2C and D) relative to their WT controls at basal levels. We observe a significant increase in mROS production in N1 HBE cell lines (<0.0001) and WT BHK cells (p = 0.004) exposed to rotenone, respectively. Rotenone further exacerbates ROS production in C1 HBE cell lines (p < 0.0001) and ΔF BHK cells (p = 0.0059) (Fig. 2A–D). Complex III inhibitor, antimycin A significantly increased ROS production in N1 HBE cell lines (p = 0.0176) and WT BHK cells (p < 0.0001), and like rotenone, this increase was heightened in C1 HBE cell lines (p < 0.0001) and ΔF BHK cells (p = 0.0056) but to a lesser degree than rotenone. Treatment with complex II inhibitor, DMM differentially impacts mROS in the CF cell lines with a significant decrease observed in ΔF BHK cells (p = 0.0079) but not in C1 HBE cell lines. These data suggest that mROS is being produced predominantly, via forward electron transport.

### 3.3 ETI and calcium modulator, BAPTA-AM reduce oxygen consumption rate in CF cell lines

**Fig. 3:**
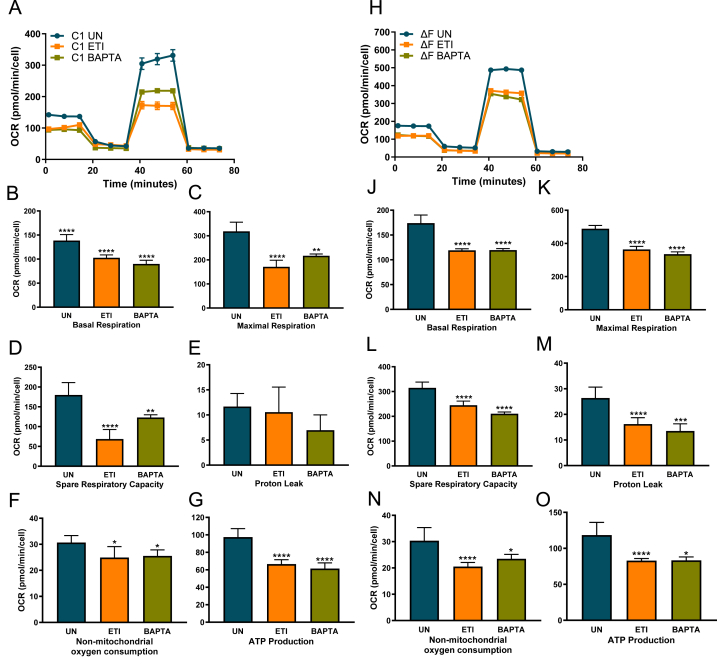
CFTR Modulators, ETI and calcium modulator, BAPTA-AM reduce OCR in CuFi1 F508del/F508del cells. OCR levels were measured in C1 HBE cell lines (A-G), and ΔF BHK cells (H-O). Cells were cultured under basal conditions and incubated in the presence or absence of ETI. Untreated cells were then cultured with BAPTA-AM. Basal respiration (B, K), maximal respiration (C, K), spare respiratory capacity (D, L), proton leak (E, M), non-mitochondrial oxygen consumption (F, N) and ATP production (G, O) were all measured. These graphs show three independent experiments with 3–6 technical replicates in each experiment for each cell line and each condition. I, ΔF BHK cells incubated in the presence of absence of ETI were immunoblotted for CFTR. All values were calculated as described in the methods. All data are presented as mean ± SEM. (∗). Statistical comparisons were performed by one-way ANOVA, followed by Tukey’s multiple comparison test, ∗p < 0.05, ∗∗p < 0.01, ∗∗∗p < 0.001, ∗∗∗∗p < 0.0001.

In both CF cell lines (C1 HBE cell lines and ΔF BHK cells) treatments with ETI therapy lowers OCR parameters to N1 or WT levels respectively, or below and are comparable to treatments with BAPTA-AM and thapsigargin (Fig. 3). Significant reductions were observed in basal respiration (C1 ETI: p < 0.0001; ΔF ETI: p < 0.0001), maximal respiration, (C1 ETI: p < 0.0001; ΔF ETI: p < 0.0001), spare respiratory capacity (C1 ETI: p < 0.0001; ΔF ETI: p < 0.0001) ATP production (C1 ETI: p < 0.0001; ΔF ETI: p < 0.0001) and non-mitochondrial oxygen consumption (C1 ETI: p = 0.0146; ΔF ETI: p < 0.0001) (Fig. 3). Proton leak was only reduced with ETI and BAPTA-AM in BHK ΔF cells (p < 0.0001), with no significant changes in C1 HBE cell lines (Fig. 3E–L).

### 3.4 Mitochondrial ROS is reduced in CF cell lines following ETI therapy

**Fig. 4:**
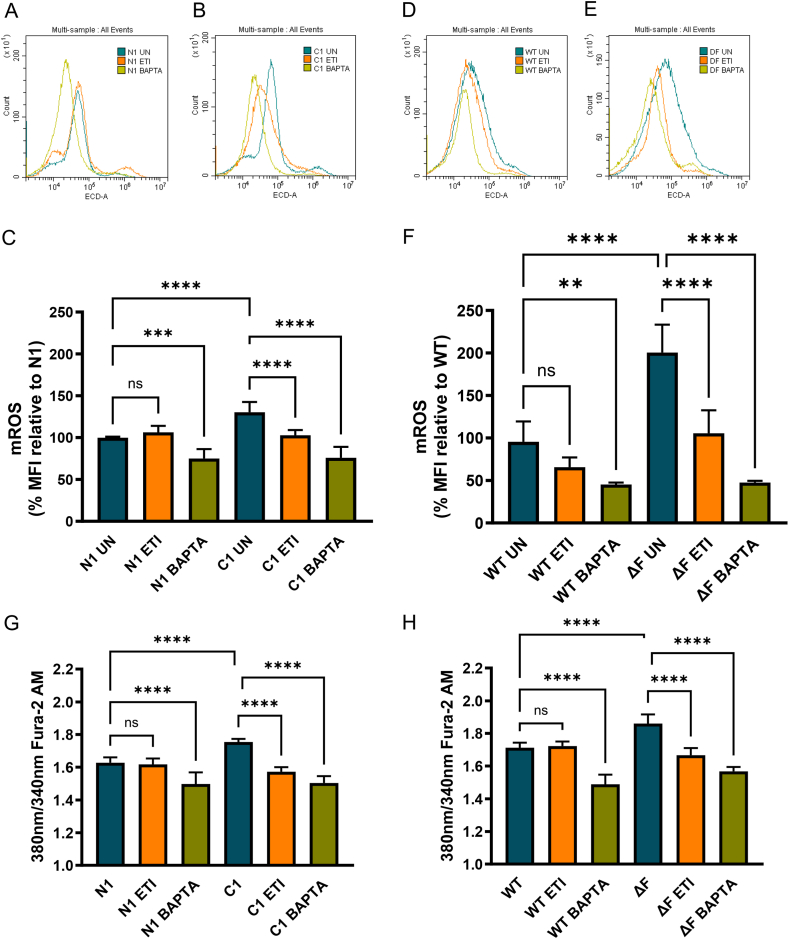
Mitochondrial ROS and calcium levels is reduced in HBE cell lines and BHK cells expressing F508del/F508del CFTR following BAPTA-AM and ETI therapy. Mitochondrial ROS was measured in N1 HBE cell lines C1 HBE cell lines (A, B, C), WT BHK and ΔF BHK (D, E, F). For all experiments, cells were incubated in presence or absence of ETI or BAPTA-AM. For mitochondrial ROS, cells were subsequently incubated with MitoSOX Red mitochondrial ROS measured by flow cytometry. For intracellular calcium assays, cells were incubated with Fura-2 AM before being incubated in BAPTA-AM. Following incubation, the fluorescence was measured with excitation parameters (Lm1 340 nM, Lm2 380 nM) and emission parameters (Lm1 510 nm, Lm2 620 nm) being set. A ratio of Lm1/Lm2 was calculated and an endpoint reading taken after 5 min recording. These graphs show three independent experiments with 3–6 technical replicates in each experiment for each cell line and each condition. All values were calculated as described in the methods. All data are presented as mean ± 95 % CI. (∗). Statistical comparisons were performed using a one-way ANOVA followed by Tukey’s multiple comparison test, ∗p < 0.05, ∗∗p < 0.01.

Next, we wanted to explore whether calcium modulators and ETI treatment reduced mROS. In the control cell line N1 HBE cell line, ETI did not alter mROS whereas N1 HBE cell lines treated with BAPTA-AM, mROS was significantly reduced (BAPTA-AM: p = 0.0002) (Fig. 4A–C). Consistent with the HBE cell lines, control cell line, BHK WT and BAPTA-AM reduced mROS significantly (BAPTA-AM: p = 0.0010) (Fig. 4D–F). In the C1 HBE cell lines and BHK ΔF cells, the increased mROS is significantly reduced by BAPTA-AM (C1 BAPTA-AM: p < 0.0001; ΔF BAPTA-AM: p < 0.0001). ETI therapy reduces mROS in both cell lines to WT levels for each cell type (C1 ETI: p < 0.0001; ΔF ETI: p < 0.0001). This data shows that ETI therapy reduces mROS in CF cell types.

### 3.5 ETI therapy decreases intracellular calcium levels in CF cell lines

To establish whether intracellular calcium levels are altered in CF cells relative to WT, we measured intracellular calcium levels using Fura-2/AM. In both cell types (HBE cell lines and BHK cells), the presence of F508del/F508del CFTR triggers increased intracellular calcium levels (C1 p < 0.0001; ΔF: p < 0.0001) (Fig. 4G and H). To establish whether ETI is directly impacting intracellular calcium levels, we compared ETI treatment with BAPTA-AM in WT and CF cell types. In N1 HBE cell line and WT cell lines, BAPTA-AM significantly reduces calcium levels (N1, p < 0.0001; ΔF p < 0.0001), whereas ETI has no impact. In C1 HBE cell line and ΔF BHK cells, BAPTA-AM reduces calcium to similar levels as N1 HBE cell line and WT BHK treated with BAPTA-AM (C1 BAPTA-AM vs C1 untreated: p < 0.0001; ΔF BAPTA-AM vs ΔF untreated: p < 0.0001). ETI also reduces intracellular calcium levels in C1 HBE cell line and ΔF BHK cells to levels, similar to untreated N1 HBE cell line and WT BHK (C1 ETI vs C1 untreated: p < 0.0001; ΔF ETI vs ΔF untreated: p < 0.0001). These findings suggest that ETI modulates mitochondrial function and mROS via mechanisms involving changes in intracellular calcium levels.

## 4 Discussion

An increase in BMI is often associated with ETI therapy and is likely to reflect improved clinical stability and physiological changes following partial CFTR correction [17,18,22,23]. This drug-associated increase in weight is predominantly a positive clinical outcome, alongside improved lung function, reduced pulmonary exacerbations and sweat chloride concentration [18]. However, excessive weight gain may affect body image, self-esteem, drug adherence and trigger future long and short term complication if overweight or obese [24].

Understanding the aberrations and impact of ETI therapy on cellular metabolism in CF provides real opportunities to further study energy requirements in the post modulator era, assess effectiveness of future therapies at normalising mitochondrial metabolism as well as the future role of diet, ageing and age related metabolic diseases on mitochondrial physiology in CF [25].

In this study, we demonstrated defective mitochondrial respiration with increased OCR in two different cell types expressing the CF F508del/F508del homozygous mutation. This increased mitochondrial respiration correlated with increased mROS. Both oxygen consumption and mROS were reduced by ETI as well as BAPTA-AM. The intracellular calcium indicator dye, Fura-2 AM revealed normalisation of Ca^2+^ levels in CF cells following ETI, directly correlating CFTR dysfunction with disrupted Ca^2+^ homeostasis. While the effect of ETI on OCR may be attributable to alterations in calcium homeostasis, the similar ETI and bapta responses do not establish a direct causal link.

We have previously observed a similar reduction in OCR with ivacaftor/tezacaftor and ivacaftor in CuFi-1 and CuFi-4 cell lines respectively (Supplementary Fig. 1). Further studies investigating the relative and dose responses of individual, combined and newer CFTR modulators would shed further light on the underlying mechanisms driving alteration in mitochondrial respiration. Alternative models using differentiated airway epithelial cells grown in an air-liquid interface may have proved more reflective of a normal physiological state. Cells differentiate at air liquid interface can result in a shift to greater oxidative metabolisms compared to those cultured undifferentiated under media [26]. We were reassured to see that both cell lines in our study produced similar results, under similar culture conditions.

While intracellular calcium accumulation is fundamental to the CF phenotype across all reported cell types [4,7,8], there are discrepancies in relation to reported mitochondrial activity and cellular oxygen consumption. Under physiological conditions, elevated Ca^2+^ stimulates the TCA cycle and oxidative phosphorylation, and consequently triggers mROS output by making the mitochondria work faster and consume more O_2_ [27]. Indeed, metabolic rate correlates well with mROS generation, suggesting that a faster metabolism simply results in more respiratory chain electron leakage [28]. Under the conditions used in this study, and consistent with previous studies, increased mitochondrial metabolism was present in HBE cell lines and BHK cells expressing the CFTR F508del/F508del mutation, when assessing basal, maximal, spare respiratory capacities and ATP production as well as mROS production. Increased ATP levels, generated through the electron transport chain, may be required in C1 HBE cell lines and ΔF BHKs because of their defective cellular processes, such as upregulation of Na^+^/K^+^ ATPase pump [29,30]. Furthermore, inhibition of the Na^+^/K^+^ ATPase in CF nasal epithelial cells has been shown previously to reduce OCR [30].

Within the physiological parameters, Ca^2+^ can stimulate nitric oxide synthase (NOS) which generates NO. It has been suggested that a major physiological role for NO is in the regulation of mitochondrial ROS output [27,31]. This signalling axis is thought to act within a physiological window of NO concentrations and pathological levels can be detrimental to mitochondrial ATP synthesis whereby NO inhibits complex IV, which would enhance ROS at CoQ. High NO in conjunction with high mitochondrial calcium levels can inhibit complex I [32]. Atlante et al., in 2016, reported impaired complex I and IV activity in CF bronchial epithelial cells, (CFBE41o) [13], changes which have reflected pathological Ca^2+^ levels with downregulation of ETC activity and increased mROS thorough alternative mechanisms.

In the presence of infection, stimuli-specific metabolic profiles, particularly in monocytes/macrophages, can differentially affect mitochondrial activity [33]. Monocytes activated with low dose LPS triggers increases in oxidative phosphorylation and glycolysis to the same degree as Pam3CysSK4 (PC3), but high doses of LPS results in a metabolic switch, known as the Warburg effect, with a shift from oxidative phosphorylation to glycolysis [33]. This is consistent with our previous work where monocytes stimulated with low dose LPS show increased oxidative phosphorylation, and, although only a trend increase in glycolysis was observed, the ability of these cells to convert to a glycolytic profile was augmented through increases in glycolytic capacity and reserve [12]. Macrophages infected directly with *B. cenocepacia* show a shift in their metabolic profile with decreased oxidative phosphorylation and glycolytic shift [34].

Both infection and inflammation drive differing cellular perturbation in CF cells with LPS and viral infections being associated with an exaggerated inflammatory response [[35], [36], [37], [38]]. Infection and inflammation is also known to drive the production of mROS, and alter Ca^2+^ mobilisation as well as mitochondrial biogenesis with LPS stimulation being associated with elevated OCR in endothelial cells [[39], [40], [41], [42]]. For this reason, we specifically focused on assessing the impact of ETI on OCR at baseline, in differing cell lines. Future studies focusing on the impact of various cofounding factors which may influence OCR as well as the potential differences in mitochondrial respiration in different organ specific cells, such as gut and pancreatic derived cell lines, are warranted.

In the present study we used two different CF cell types in the absence of infection. The ΔF BHK cells showed upregulated oxidative phosphorylation, suggesting that in the absence of any infection or inflammatory stimuli, CFTR dysfunction alone influences the metabolic trajectory of a particular cell type. Previous studies have demonstrated elevation in ER Ca^2+^ concentration in homozygous F508del-CFTR expressing (CFBE41o-) compared to WT CFTR expressing (16HBE14o) bronchial epithelial cells [7]. This difference could be normalised by the CFTR corrector, VX-809 [7]. The retention of F508del-CFTR within the ER increases SERCA (Sarcoplasmic/Reticulum Ca2 + ATPase) in CF cells compared to corrected CF cells (VX-809) and non-CF cells [7] consistent with our studies which show that intracellular Ca^2+^ levels are normalised with partial CFTR correction. A similar effect on calcium homeostasis has been reported in transfected Chinese hamster ovary (CHO-K1) cells and adenocarcinomic human alveolar basal epithelial (A549)/malignant melanoma (A375) cells exposed to VX-770 and VX-809 respectively [43,44]. Similarly VX-809 has been associated with a reduction in mROS production [44].

The rescue of F508del-CFTR by ETI has previously been explored in primary airway epithelial cells, as well as CF bronchial epithelial cells and BHK expressing F508del-CFTR (as used in our study). They found that ETI was efficient at rescuing F508del-CFTR-abnormal maturation, apical membrane localisation and function [19].

In conclusion, our study demonstrates that ETI downregulates the levels of basal, maximal, spare respiratory capacity and ATP production in two CF cells lines to WT levels or below. These changes are likely to reflect dysregulation of calcium signalling, a consequence of CFTR dysfunction.

## CRediT authorship contribution statement

**H.H. Jarosz-Griffiths:** Writing – review & editing, Writing – original draft, Visualization, Validation, Software, Resources, Project administration, Methodology, Investigation, Formal analysis, Data curation, Conceptualization. **L.R. Caley:** Writing – review & editing, Writing – original draft, Methodology. **S. Lara-Reyna:** Writing – review & editing, Resources, Methodology. **S. Savic:** Writing – review & editing, Supervision, Resources. **I.J. Clifton:** Writing – review & editing, Supervision. **M.F. McDermott:** Writing – review & editing, Supervision, Resources. **D.G. Peckham:** Writing – review & editing, Writing – original draft, Supervision, Resources, Project administration, Methodology, Investigation, Funding acquisition, Data curation, Conceptualization.

## Data availability statement

Data included in article/supp. material/referenced in article. Further data will be made available on request to the author, Dr Jarosz-Griffiths.

## Funding

This work was supported by Leeds Hospitals Charity, Cystic Fibrosis Trust Strategic Research Centre grant (SRC009), the CONACyT and by a charitable donation from Gary Shuckford.

## Declaration of competing interest

The authors declare that they have no known competing financial interests or personal relationships that could have appeared to influence the work reported in this paper.
