# Supplementary figures and images for: Heightened mitochondrial respiration in CF cells is normalised by triple CFTR modulator therapy through mechanisms involving calcium
Source: Heliyon. 2024 Oct 11;10(20):e39244. doi: 10.1016/j.heliyon.2024.e39244 (PMC11532250; doi:10.1016/j.heliyon.2024.e39244)

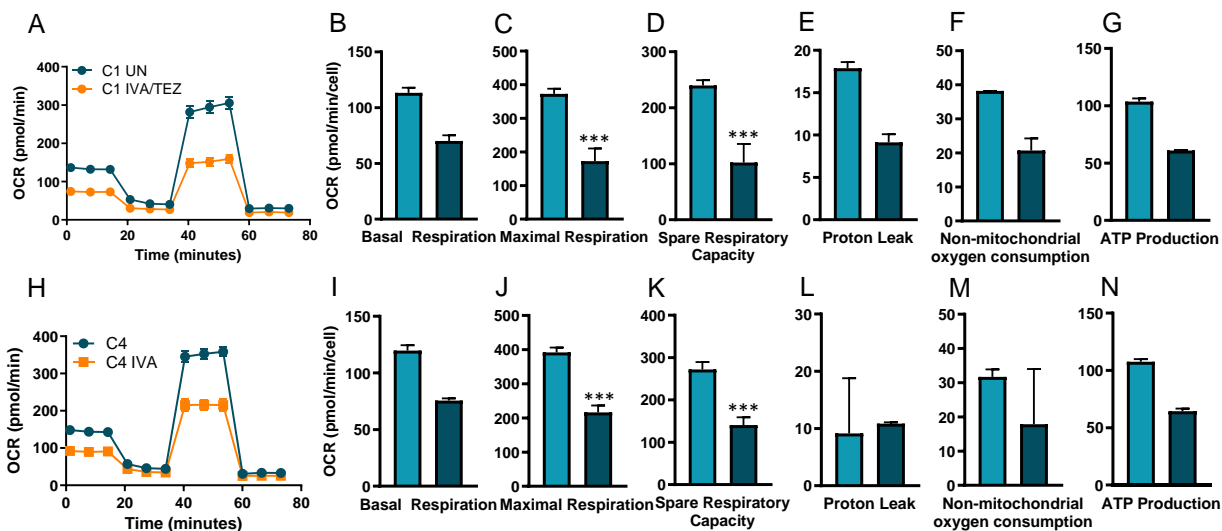

Supplement: Multimedia component 1 [file mmc1.pdf]
